# On‐Chip Biogenesis of Circulating NK Cell‐Derived Exosomes in Non‐Small Cell Lung Cancer Exhibits Antitumoral Activity

**DOI:** 10.1002/advs.202003747

**Published:** 2021-01-28

**Authors:** Yoon‐Tae Kang, Zeqi Niu, Thomas Hadlock, Emma Purcell, Ting‐Wen Lo, Mina Zeinali, Sarah Owen, Venkateshwar G. Keshamouni, Rishindra Reddy, Nithya Ramnath, Sunitha Nagrath

**Affiliations:** ^1^ Department of Chemical Engineering Biointerfaces Institute University of Michigan Ann Arbor MI 48109 USA; ^2^ Rogel Cancer Center University of Michigan 1500 East Medical Center Drive Ann Arbor MI 48109 USA; ^3^ Michigan Medicine Pulmonary and Critical Care Division University of Michigan Ann Arbor MI 48109 USA; ^4^ Michigan Medicine Thoracic Surgery Clinic Taubman Center 1500E Medical Center Dr. SPC 5344 Ann Arbor MI 48109 USA; ^5^ Department of Internal Medicine University of Michigan Ann Arbor MI 48109 USA

**Keywords:** cancer immunotherapy, circulating tumor cells, exosome biogenesis, microfluidics, natural killer cells

## Abstract

As the recognition between natural killer (NK) cells and cancer cells does not require antigen presentation, NK cells are being actively studied for use in adoptive cell therapies in the rapidly evolving armamentarium of cancer immunotherapy. In addition to utilizing NK cells, recent studies have shown that exosomes derived from NK cells also exhibit antitumor properties. Furthermore, these NK cell‐derived exosomes exhibit higher stability, greater modification potentials and less immunogenicity compared to NK cells. Therefore, technologies that allow highly sensitive and specific isolation of NK cells and NK cell‐derived exosomes can enable personalized NK‐mediated cancer therapeutics in the future. Here, a novel microfluidic system to collect patient‐specific NK cells and on‐chip biogenesis of NK‐exosomes is proposed. In a small cohort of non‐small cell lung cancer (NSCLC) patients, both NK cells and circulating tumor cells (CTCs) were isolated, and it is found NSCLC patients have high numbers of NK and NK‐exosomes compared with healthy donors, and these concentrations show a trend of positive and negative correlations with bloodborne CTC numbers, respectively. It is further demonstrated that the NK‐exosomes harvested from NK‐graphene oxide chip exhibit cytotoxic effect on CTCs. This versatile system is expected to be used for patient‐specific NK‐based immunotherapies along with CTCs for potential prognostic/diagnostic applications.

## Graphical abstract

Natural killer (NK) cells are lymphocytes that have the ability to destroy cancer cells without antigen presentation. The NK cell‐derived exosomes exhibit higher stability, greater modification potentials, and less immunogenicity compared to NK cells. Here, a novel microfluidic system to collect patient‐specific NK cells and on‐chip biogenesis of NK‐exosomes for diagnostic and therapeutic use of NK‐exosomes is proposed.

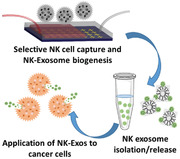

## 1 Introduction

Cancer is the leading cause of death worldwide, and mortality from cancer remains high despite significant progress in cancer treatments. Cancer immunotherapy has emerged as a promising strategy to treat cancers, most notably by engaging the adaptive immune system (e.g., immune checkpoint inhibitors) or through cell‐based approaches (e.g., adoptive T cell therapies, natural killer (NK) cell therapies) involving the innate immune system.^[^ ^1^, ^2^, ^3^ ^]^ The U.S. Federal Drug Administration (FDA) has approved several of these therapies, such as pembrolizumab and ipilimumab (immune checkpoint inhibitors), for both solid organ and hematological malignancies in the past decade, revolutionizing treatments of many cancer that were considered incurable in the past.^[^ ^4^ ^]^

Specifically, chimeric antigen receptor (CAR) T‐cell therapy, which uses T cells genetically engineered to display receptors on their surface called chimeric antigen receptors, was approved by the FDA for treatment of specific refractory leukemias.^[^ ^5^, ^6^ ^]^ Despite these successes, T cell‐based therapies are handicapped by factors including an expensive, time‐consuming process of engineering and expanding T cells, as well as efficacy limitations arising from low major histocompatibility complex (MHC) expression on tumor cells.^[^ ^7^ ^]^ To circumvent these issues, other approaches have been proposed, which involve the study of other members of the innate immune system, such as NK cells.

NK cells are lymphocytes that can be cytotoxic against a wide range of cells, having the ability to destroy infectious as well as transformed cells without antigen presentation.^[^ ^8^, ^9^ ^]^ The high cytotoxicity of NK cells against circulating tumor cells (CTC), which are considered as the seed of metastasis, was previously reported.^[^ ^10^ ^]^ Because of the broad cytotoxicity and rapid reaction, adoptive NK cell therapy aimed at increasing NK cell numbers may be a promising immunotherapeutic approach. Compared with T cell‐based therapies, NK cell therapies are independent of antigen presentation and can be better controlled to reduce the risk of cytokine storms.^[^ ^11^ ^]^ Thus far, over 150 NK cell‐based immunotherapy clinical trials have been performed and applied to various cancers including ovarian, breast,^[^ ^12^ ^]^ and non‐small cell lung cancer (NSCLC).^[^ ^13^ ^]^ Recently, NK cell therapies have shown promise in relapsed/refractory acute myeloid leukemia (AML) patients who were pretreated with chemotherapy regimens.^[^ ^14^, ^15^ ^]^ A trial testing HER2‐specific CAR NK‐92 cells in glioblastoma was also launched in 2018. However, NK cell‐based therapies still struggle to overcome some of the innate limitations of cell‐based therapies, such as a lack of ability to deliver adequate numbers of NK cells to tumors because of limited targeting capabilities. For example, NK cell therapy for brain cancer is challenging, given the difficulty in crossing the blood–brain barrier (BBB) and the blood–tumor barrier (BTB).^[^ ^16^, ^17^ ^]^ To overcome these limitations while simultaneously utilizing NK cell's benefits, several newer approaches, such as utilizing NK cell‐derived exosomes or microvesicles, have recently been studied by a few groups^[^ ^18^, ^19^ ^]^ to improve NK cell‐based immunotherapy approaches.

Exosomes are a type of extracellular vesicle (EV) secreted by various cells. They are nanoscopic courier vesicles (30–150 nm) carrying lipids, nucleic acids, metabolites, and proteins, hence playing a central role in intercellular communication and macromolecule transmission. They also enable transport of proteins that convey genetic information between cells. Almost all membranous cells within the human body secrete exosomes,^[^ ^20^ ^]^ and these exosomes can mimic many of the salient features of their mother cells while also displaying their own distinguishing features. These innate unique protein compositions of exosomes allow for specific cellular uptake and target‐homing capabilities, which is differentiated from standard nanoparticle‐based drug carriers.^[^ ^21^, ^22^ ^]^ Exosomes are also characterized as biocompatible and stable in physiological conditions with a long circulating half‐life in blood.^[^ ^23^ ^]^ Therefore, exosomes are currently being considered for use as natural drug carriers or agents for cancer immunotherapy, both of which aim to curtail the spread of cancer throughout the body and retard the growth of tumor cells.^[^ ^24^ ^]^ To utilize innate merits of exosomes, exosomes have been hybridized with conventional nanoparticles to be used as a drug carrier for cancer chemotherapy.^[^ ^25^ ^]^

The unique characteristics of exosomes derived from certain immune cells such as NK cells may offer solutions to many of the challenges arising from complications of the tumor microenvironment. Their nanosize and abundance are ideal for cancer treatment via effective trafficking to the solid tumor location and infiltration into the tumor microenvironment.^[^ ^26^ ^]^ Exosomes derived from NK cells can penetrate several typically problematic barriers including the blood–brain barrier and the blood–tumor barrier, which has been relatively impenetrable to NK cells. As such, NK cell immunotherapies for cancers such as glioblastoma have been restricted in their efficacy because of reliance on small tumor‐induced BBB disruptions for immune cell uptake.^[^ ^27^ ^]^ Also, infiltration of NK cells into tumors, such as lung cancer, is largely influenced by cytokine profiles of tumor microenvironment, which sometimes impairs NK cell's activities.^[^ ^28^ ^]^

NK cell‐derived exosomes provide an efficient alternative for treatment of certain cancers. They naturally express IFN‐*γ*, FasL, and multiple cytotoxic proteins that can induce apoptosis via multiple killing mechanism.^[^ ^28^, ^29^ ^]^ Exosome's innate cell–cell transfer ability can stimulate further tumor microenvironmental activity or communicate with surrounding cells, activating their cytotoxic effects.

Recent studies have demonstrated the effectiveness of NK cell‐derived exosomes in lysing malignant tumor cells, including mediating a significant antitumor response against acute myeloid leukemia and melanoma.^[^ ^19^, ^30^ ^]^ While NK cells have shown promising results despite limited tumor access, exosomes present an option for unhindered tumor access by particles with NK cell properties and associated benefits including stability, consistency, and modification potential.^[^ ^23^ ^]^

Therefore, selective isolation of NK cells and the subsequent harvesting of exosomes is a promising approach for NK therapies in the future. However, because of the technical challenges of isolating highly pure, live populations of NK cells and the subsequent collection of exosomes derived from these cells, this approach has not been successfully implemented.^[^ ^18^ ^]^ There have been several separate attempts to optimize specific cell isolation or cell‐derived exosome collection; however, to date, there are no streamlined platforms to isolate specific subtypes of cells and to harvest specific exosomes from a heterogeneous sample.

Microfluidics and microdevices have been utilized for decades for the isolation of cells and vesicles from a complex specimen with higher efficiency and sensitivity than conventional bulky equipment‐based isolation. Microfluidic technologies for liquid biopsies have rapidly evolved and have shown success in capturing rare cells, including circulating tumor cells,^[^ ^31^, ^32^, ^33^ ^]^ stem cells,^[^ ^34^, ^35^ ^]^ and extracellular vesicles.^[^ ^36^, ^37^, ^38^ ^]^ Microfluidic devices functionalized with antibodies to capture specific cell populations are a powerful tool to achieve a high‐purity isolation with minimal resources and limited quantity of clinical samples.^[^ ^39^ ^]^ In addition, recent advances of antibody conjugation chemistry offer an advantage to selectively release isolated targets for further downstream analysis and functional studies. Several release strategies have been studied utilizing surface antibody conjugates, including cleavable cross‐linkers,^[^ ^40^, ^41^ ^]^ dissociable thermal sensitive polymer substrates,^[^ ^42^ ^]^ and methods that take advantage of competing binding affinities between biotinylated antibodies and biotin derivatives.^[^ ^43^, ^44^, ^45^, ^46^, ^47^ ^]^ Use of cleavable cross‐linkers or dissociable substrates requires special chemicals or condition changes which may be crucial to the final products or might alter the contents of the released targets. Chemical release strategies utilizing competing binding affinities such as the use of biotin to displace antibodies conjugated with biotin analogs with lower streptavidin bond preference have demonstrated notable success in complete target cleavage under mild conditions.^[^ ^43^, ^44^, ^45^, ^46^, ^47^ ^]^ However, incorporation of this chemistry into microsystems has rarely been studied.

**Figure 1:**
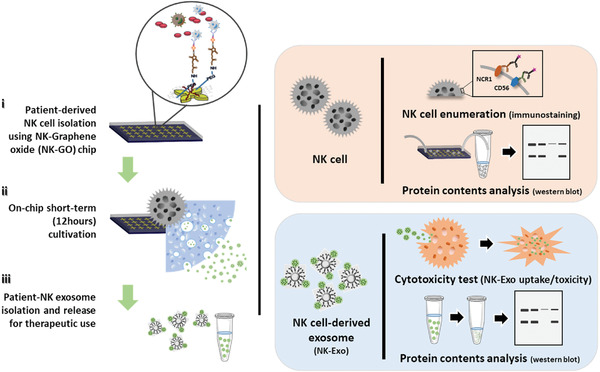
Schematic diagram of the microfluidic technology approach for on‐chip natural killer (NK) cell isolation, in situ NK cell‐derived exosome biogenesis, and recovery for potential therapeutic use of NK exosomes.

Given the combined merits of microfluidic devices and chemical release strategies, we propose a streamlined microfluidic platform to harvest NK exosomes (NK‐Exos) from viable NK cells isolated on chip (**Figure** **1**). By using a graphene oxide microfluidic chip functionalized with antibodies against natural killer cells (NK‐GO chip), we isolate NK cells on chip with high purity and viability. The deposition of 2D GO sheet has a large surface area and provides more binding sites to isolate targets compared to the identical sized device without GO deposition.^[^ ^48^ ^]^ Thanks to the biocompatibility of GO, the captured and viable NK cells undergo short‐term incubation for NK‐Exo secretion, and the recovery of the NK‐Exos is accomplished using anti‐CD63 conjugated magnetic beads (ExoBeads). For the functional study of NK‐Exos, here, instead of using irreversible binding of biotin to avidin, we used desthiobiotinylated anti‐CD63 and avidin‐conjugated beads.^[^ ^46^ ^]^ Therefore, we were able to easily strip the isolated exosomes from the beads using biotin solutions because of competing reactions between biotin and desthiobiotin against avidin.^[^ ^47^, ^49^, ^50^, ^51^ ^]^ This mild and biocompatible release process enables further nanoparticle tracking analysis (NTA) and functional studies of NK‐Exos. Using clinical blood samples from patients with NSCLC and healthy donors, we compared total NK cell number and NK exosomes in terms of concentration and size, along with their correlation with CTC number in blood. The isolated and released exosomes were further characterized using in‐house CTC‐derived cell line cytotoxicity assay to evaluate preliminary therapeutic potential. We believe our novel and versatile platform can set the stage for future work relating to NK exosome‐based cancer immunotherapy.

## 2 Results and Discussion

### 2.1 Viable NK Cell Isolation Using a NK‐GO Microfluidic Chip

**Figure 2:**
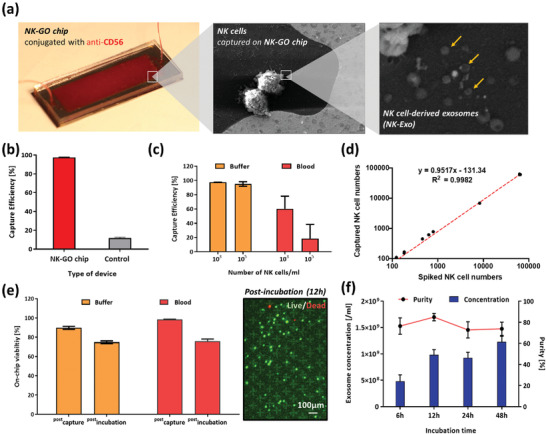
On‐chip NK cell isolation and in situ exosome biogenesis and harvesting from the isolated NK cells: a) in situ exosome biogenesis from isolated NK cells on NK‐GO chip; b) NK cell capturing performance of NK‐GO chip compared to control device without antibody conjugation; c) capture efficiency of the NK‐GO chip for different concentrations of NK cells spiked in buffer and blood samples; d) captured NK cells depending on spiked NK cells in buffer solution. The red dashed line is a linear fit to the data; e) viability of the isolated NK cells at different time points and conditions (left) and live/dead staining of isolated NK cells on chip after 12 h incubation; f) exosome secretion from NK cells depending on incubation times.

The high surface area and biocompatibility of GO nanosheet facilitates sensitive and efficient NK cell isolation and on‐chip short‐term culture for NK‐exo biogenesis. The ability of the NK‐GO chip (**Figure** **2**) to isolate NK cells was initially examined using spiked cell experiments with two different conditions: 1) NK cell line (NK‐92MI) spiked in phosphate‐buffered saline (PBS) buffer and 2) NK cell line spiked in whole blood. NK cell capturing performance of NK‐GO chip was first compared with control device without antibody conjugation and showed that the current device isolates more than 95% NK cells spiked in buffer, which implies that anti‐CD56 and its functionalization on the GO chip is capable of NK cell isolation (Figure 2b). Based on previous studies,^[^ ^52^, ^53^ ^]^ we found that the frequency of NK cells is usually in the range of ≈10^5^ cells mL^−1^ of peripheral blood, thus we examined our NK‐GO chip's NK cell capturing performance at two different concentrations, 10^3^ and 10^5^ cells mL^−1^. The prelabeled NK cells at two different concentrations were spiked into each of the two different model samples, and the number of the captured cells was enumerated using a fluorescence microscope. Capture efficiency at four different conditions are shown in Figure 2c. In each case, capture efficiencies of NK cells spiked in blood were considerably lower (30%) than that of buffer. This is expected because of the presence of NK cells in blood, leading to competition for capture at binding site on the NK‐GO chip; reported capture efficiencies are calculated based only on the number of prelabeled NK‐92MI cells. Linearity plot was also prepared based on NK cell recovered compared to NK cell spiked (Figure 2d). In addition, the viability of NK cells was evaluated to ensure that the isolated NK cells are viable and hence can exosomes during the postcapture incubation. Using the live/dead staining assay postcapture, we found that over 70% of the isolated NK cells remained viable before and after short‐term incubation (Figure 2e).

### 2.2 On‐Chip NK Exosome Biogenesis and Harvesting

To evaluate the exosome biogenesis rate from NK cells, we spiked 5000 NK‐92MI cells into a six‐well plate and incubated them for 6, 12, 24, and 48 h. After incubation, we collected the supernatant from each well and centrifuged them at 300 *g* for 10 min to remove cells or cellular debris. The supernatant was then ultracentrifuged using an Airfuge (Beckman, USA) at 100 000× *g* for 30 min to collect NK‐Exos. Exosome concentration was determined by NTA, and exosome secretion rates showed an increasing trend with increased incubation time (Figure 2f). However, exosomal purity, the fraction of exosome‐sized vesicles out of all vesicles in the sample, was highest at 12 h of incubation. It is possible that the longer incubations led to cell death and secretion of apoptotic bodies and microvesicles.^[^ ^54^, ^55^, ^56^ ^]^ Considering the low O_2_ concentration in on‐chip conditions compared to cell culture flasks, combined with the goal of establishing a rapid assay, we implemented the protocol of incubating for 12 h for on‐chip NK exosome biogenesis and then NK exosome harvesting. Following NK exosome secretion biogenesis experiments, we compared the biogenesis rate between the off‐chip (well plate) condition and the on‐chip condition (Figure S1, Supporting Information). The hourly exosome‐biogenesis rate per cell was marginally different between off and on‐chip conditions. Both biogenesis rate and purity were higher when the cells secreted exosomes in on‐chip conditions, which implies that the present short‐term on‐chip culture of isolated NK cells on chip is feasible for exosome harvest using clinical samples. The overall NK exosome biogenesis from isolated NK cells was also analyzed using scanning electron microscope (SEM) (Figure 2a), showing that viable isolated NK cells on‐chip secrete exosome‐like vesicles.

### 2.3 NK Cell‐Derived Exosome Harvest/Recovery by ExoBead

**Figure 3:**
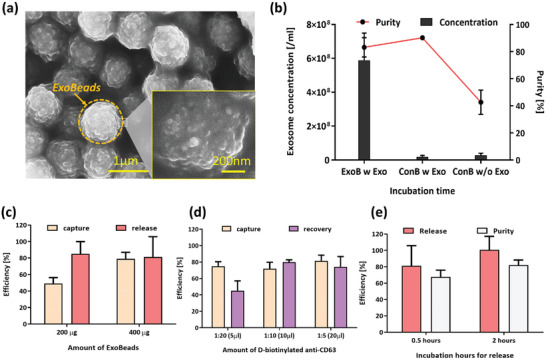
ExoBead‐based NK exosome isolation and release for therapeutic use: a) scanning electron microscope image of the isolated exosomal vesicles on ExoBeads with supernatant from NK‐GO chip after 12 h incubation; b) concentration and purity of exosomal vesicles recovered from NK‐92MI culture supernatant under three different conditions using ExoBeads (ExoB) and control beads (ConB) nonconjugated with antibodies; c) capture and release performance of ExoBeads depending on amounts of beads for identical volume of NK‐GO chip supernatant; d) capture and recovery performance of ExoBeads depending on amounts of d‐biotinylated anti‐CD63 during antibody conjugation; e) release and purity performance comparison between two different incubation times with biotin solution for exosome release.

Using the supernatant from the NK cells isolated on NK‐GO chips, we further isolated the exosomes selectively using our ExoBeads (**Figure** **3**). Beads without an antibody (anti‐CD63) conjugation were prepared as a control (control beads). To initially evaluate the exosomal recovery performance, we prepared the following three different conditions: a) ExoBeads with on‐chip NK cell supernatant sample, b) control beads with on‐chip NK cell supernatant sample, and c) control beads without supernatant sample. Using these conditions, we isolated and released the bound vesicles from beads and evaluated their concentration by NTA. As a result, we verified that only the sample prepared with ExoBeads (a) had any detectable amount of exosomal vesicles, with more than 83% of purity (Figure 3b). Sample from condition (b) had the highest purity, but its exosomal concentration was considerably lower than that from condition (a). After this quantitative study, we imaged the ExoBeads after capturing exosomes from the on‐chip NK cell supernatant samples using SEM. The SEM images of the ExoBeads clearly showed that the beads isolated exosomal vesicles. The sizes of these vesicles ranged 80–130 nm (Figure 3a). Given the specific antibody used for exosome capture and the size criteria we applied to the resultant, we concluded that our ExoBeads are capable of isolating exosome‐like vesicles from heterogeneous samples containing other subtypes of extracellular vesicles, such as microvesicles and apoptotic bodies.

To further optimize NK exosome isolation and release, several conditions were varied to test their effects on the performance of the ExoBeads. These conditions include initial bead amount, concentration of d‐biotinylated antibody reagents, and incubation time with biotin for release. For these optimization experiments, we prepared the purified NK‐92MI exosomes using ultracentrifugation of NK cell supernatant, and the identical concentration of NK exosomes was used for all experiments. The optimization was evaluated in terms of capture efficiency, release efficiency, recovery rate, and purity. The definitions of those terminologies are summarized in Section S1 (Supporting Information).

Having previously demonstrated the capture capabilities of the anti‐CD63 conjugated beads compared to control beads, we this time looked to identify the optimal concentration of beads to use for isolation. NK exosome isolation and release were evaluated using both 200 µg (20 µL) and 400 µg (40 µL) of magnetic beads conjugated with d‐biotin anti‐CD63 (Figure 3c). Here, we found a marked difference in capture efficiency between the 200 and 400 µg CD63 bead samples showing a 30% higher capture efficiency while doubling the bead amount. This increase in capture efficiency with higher bead concentration is likely because of exosome saturation of the anti‐CD63 bonding sites along the beads surface when using 200 µg of beads. While there is a notable difference in capture efficiency between the 200 and 400 µg conjugated bead samples, there is only a 3.91% difference in release efficiency between the two. The minimum release efficiency value between the two conjugated bead volumes is 81.24%, indicating that the binding affinity between biotin in the release buffer and the streptavidin on the beads is significantly stronger than that of d‐biotin and streptavidin. The 50 × 10^−3^ m biotin solution used to flush out the captured exosomes easily extricated the d‐biotin from its bond with streptavidin, releasing the exosome–biotin complex into solution. These high capture and release efficiencies demonstrated the possibility of therapeutic use of NK exosomes.

We further evaluated the effect of d‐biotinylated anti‐CD63 dilution ratio for capturing exosomes. We hypothesized that increased levels of d‐biotin may lead to greater exosomal capture efficiencies. 40 µL of ExoBeads were prepared with 1:5, 1:10, and 1:20 dilution ratios of d‐biotinylated anti‐CD63. All experiments were performed using 200 µL of pure NK exosome stock solution with 1% bovine serum albumin (BSA). Results for capture and recovery efficiency are shown in Figure 3d. These results show high capture efficiencies of >70% for all concentrations of d‐biotin tested. The 1:20 d‐biotin dilution ratio samples showed significantly decreased recovery rate compared to other tested dilutions. This might be because of saturation of the streptavidin binding sites at d‐biotin concentrations of 1:10 and 1:5 (saturation at MR = 4, while 10–20% of these sites nonspecifically bind). Saturation of the streptavidin binding sites by d‐biotin forces biotin to replace d‐biotin in the binding site, as opposed to binding at unbound sites, leading to greater release of the CD63 bound exosome. This also accounts for the associated increase in purity. As capture, release, and purity performance is almost identical between the 1:5 and 1:10 d‐biotin solutions, we determined the 1:10 antibody dilution ratio to be optimal for further experiments.

d‐biotin‐based reversible reactions have been incorporated into various platform,^[^ ^49^, ^50^, ^51^ ^]^ and we have optimized this reaction to our NK exosome capture and release. d‐biotin is released from the beads using the binding affinity disparity between d‐biotin and biotin with streptavidin. In attempting to maximize release output, we believed biotin release incubation times may influence the amount of d‐biotin released from beads. This is supported by previous work showing biotin analogs, including desthiobiotin, demonstrate more complete release with longer corresponding biotin incubation.^[^ ^43^, ^44^, ^45^ ^]^ Biotin release incubation times of 0.5 and 2 h were tested, and the results for both incubation lengths are shown in Figure 3e. These results show that an increased biotin incubation time led to roughly 20% more exosomes being released from the ExoBeads. Exosomal purity in the released samples showed a similar increase with prolonged incubation. This is likely because of the biphasic nature of d‐biotin and biotin exchange reactions, as described by Hirsch et al.^[^ ^46^ ^]^ Up to 90% of d‐biotin–streptavidin bonds can be expected to break within minutes of introduction of biotin solution. However, a portion of the initial d‐biotin solution will form stronger bonds with the biotin‐binding protein (streptavidin on the ExoBeads) and in the presence of biotin dissociate at a significantly slower rate, taking roughly 2 h for a complete exchange.^[^ ^46^ ^]^ Incubation times beyond 2 h were not tested, with high release efficiencies obtained from a 2 h incubation deemed to be satisfactory.

### 2.4 Molecular Characterization and Cytotoxic Capabilities of NK Cells and NK‐Exos Using the Present Microsystem

**Figure 4:**
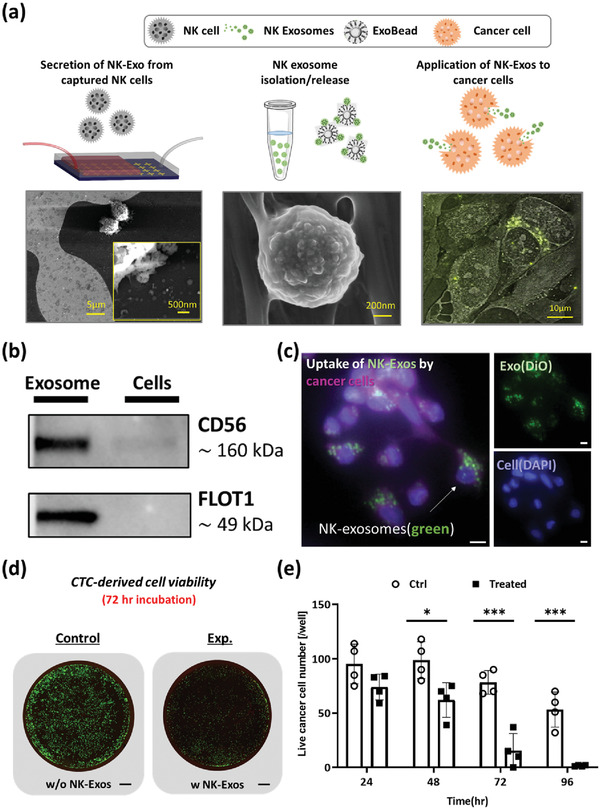
Cytotoxicity of NK cell‐derived exosomes: a) NK cells (left) and NK exosomes (center) recovered from current platform and theranostic use of NK exosomes with cancer cells (right); b) western blots showing the positive expression of CD56 and FLOT1 in exosome lysate using a NK‐92MI spike in buffer sample for on‐chip exosome biogenesis. The cell lysate from the same device also show positive expression for CD56; c) uptake of NK‐92MI exosomes by in‐house CTC‐derived cell line. Exosomes are fluorescently labeled in green (FITC) channel, and cell membrane is labeled in violet (Cy5) (Scale bar *=* 10 µm); d) cytotoxicity comparison between control well and NK exosome treated well at 72 h (Scale bar = 800 µm); e) in vitro cytotoxicity experiment using exosomes derived from NK‐92MI. Live cells were quantified through a live/dead assay that was performed 24, 48, and 72 h after treating cancer cells with or without NK exosomes. Unpaired *t*‐tests (two‐tailed) were used to compare the differences between live cell count between NK exosome treated (*n* = 4) versus control (*n* = 4). Asterisks denote one of three levels of statistical significance (*p ≤ 0.05; **p ≤ 0.01; ***p ≤ 0.001).

To demonstrate a streamlined microfluidic approach to harvest NK‐Exos from clinical samples, we first optimized our platform using NK cell model sample having NK cells spiked in blood (**Figure** **4**a). The NK cell model samples were processed until the PBS wash step following our optimized conditions. After PBS wash and application of 300 µL of serum‐free culture media, the devices were then transferred into 37 °C, 5% CO_2_ incubator for exosome secretion. After short‐term incubation, on‐chip supernatant was collected for exosome isolation by flowing serum‐free media into the device. Following exosome harvesting, we ran 4% paraformaldehyde (PFA) or radioimmunoprecipitation assay buffer (RIPA buffer) through the device to fix or lyse captured cells on chip, respectively. Immunofluorescence (IF) and western blot were performed to characterize the NK cells captured on chip, whereas NK exosomes were characterized by western blot. In addition, NK exosomes were investigated for cellular uptake and potential cancer cell cytotoxicity. On‐chip staining of captured cells after fixation was conducted using our optimized NK staining panel including CD56 and NCR1 (Section S3, Supporting Information). The CD56 and NCR1 combination displayed positive fluorescence on NK‐92MI (Figure S2, Supporting Information) but negative on Jurkat cells (Figure S3, Supporting Information). This staining panel was used thoroughly to identify and enumerate NK cell number from limited number of T cell contamination.

A direct comparison of CD56 and FLOT1 expression between captured cell lysate and ExoBead lysate was evaluated by western blot analysis (Figure 4b) using a NK‐92MI spike sample. Previously, exosomes derived from NK cells were shown to express NK cell signature protein (CD56),^[^ ^18^ ^]^ exosomal protein (ALIX, CD63),^[^ ^19^ ^]^ and lytic protein (FasL and Perforin).^[^ ^57^ ^]^ We chose two protein markers, CD56 and FLOT1, for western blot analysis. From our study, CD56 is expressed in both cell lysate and ExoBead lysate, with higher expression in our ExoBead lysate. Furthermore, FLOT1, an exosomal marker, is expressed in isolated ExoBead lysate sample but not cell lysate, which is in alignment with previous studies.

To further evaluate NK‐Exo uptake by cancer cells, we prepared cancer cells from our in‐house patient‐derived expanded CTC line^[^ ^58^ ^]^ and exosomes from NK‐92MI cells. The prepared cells and exosomes were stained using Cellmask and DiO, respectively, and incubated together for 3 h. After 3 h, NK cell‐derived exosomes showed colocalization with cancer cells and within cancer cell membrane (Figure 4c). Long‐term in vitro high‐resolution live image also showed an uptake of NK‐Exo by cancer cells (Figure S4, Supporting Information), which implies our NK‐Exos are taken up by cancer cells. Furthermore, we evaluated the potential cytotoxicity of NK‐92MI derived exosomes. Live/dead assay was conducted at 24, 48, and 72 h individually, then live cells (stained green) were enumerated. A comparison between the control and NK‐Exo treatment wells was shown by immunofluorescence scanning of the whole well (Figure 4d), with green fluorescence indicating live cells. In all cases, cell number in control wells increased because of cell proliferation, whereas in the NK exosome‐treated well, the cancer cells showed a peak number at 48 h but subsequently decreased and dead after 72 h (Figure 4e). Thus, 72 h incubation time was applied for preclinical cytotoxicity experiments using NK‐Exos from patients. Further NK‐Exo cytotoxicity experiments are described in Section S5 (Supporting Information).

From this model sample experiment, we identified NK cells on chip using a DAPI+/CD56+/NCR1+ NK cell panel, showed that the recovered NK‐Exos from NK cells express CD56 and FLOT1, and that they are able to be taken up by cancer cells and result in cytotoxic cell death of the cancer cells.

### 2.5 Preclinical Study of NK Cell/Exosome Using Clinical Specimens

**Figure 5:**
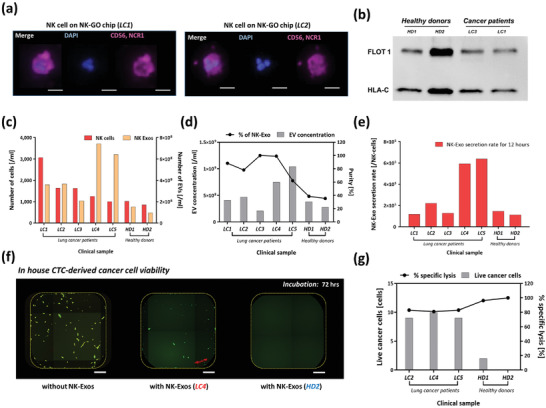
Analysis of clinical samples from NSCLC patients using NK‐GO microfluidic platform: a) immunofluorescence image examples of CD56/NCR1 + NK cells captured on NK‐GO chip (Scale bar = 20 µm); b) western blot analysis for showing the positive expression of FLOT1 and HLA‐C in exosomes from clinical samples; c) profiling in quantity of NK cells and NK cell‐derived exosomes among different patients and healthy individuals observed after 12 h on‐chip incubation; d) total extracellular vesicle concentration and percentage of exosomes among patient samples and healthy control samples; e) biogenesis of exosomes quantified as secretion rate of exosomes per captured NK cells for 12 h; f) Live cancer cell number after 72 h incubation and percentage of specific lysis between samples from cancer patients and healthy donors (Scale bar = 20 µm); g) cytotoxicity of clinical sample driven NK‐Exos to CTC‐derived cells after 72 h incubation.

Following experiments with model samples, we validated our approach with whole blood samples collected from NSCLC patients (*n* = 5) and healthy donors (*n* = 2). We used 1–2 mL whole blood sample for each experiment. Whole blood samples were processed through NK‐GO chip thoroughly and NK‐Exos were harvested from the isolated NK cells. **Figure** **5**a shows NK cells from two NSCLC cancer patients stained by the predefined NK staining panel.

Using four clinical samples including cancer and healthy donors, we analyzed the exosomal protein expression in NK cell‐derived exosomes. The clinical samples, both healthy and patient, indicate sufficiently high levels of FLOT1 and HLA‐C after isolation using anti‐CD63 magnetic beads (ExoBeads) (Figure 5b). In combination, these bands indicate the presence of EVs isolated from our target NK cells.

The cells captured by NK‐GO chip expressed CD56 and NCR1 dominantly. The number of NK cells, NK cell‐exosome quantity, and their cytotoxicity were evaluated using fluorescent staining, NTA, and in vitro cytotoxicity test, respectively. Cancer patients showed a higher number and concentration of NK cells and NK exosomes, respectively, compared to two healthy donors (Figure 5c). Cancer patients showed higher proportion of NK exosomes among total EV concentration as well (Figure 5d). Thus far, various studies showed no significant difference in overall NK cell concentration in peripheral blood of patients with various forms of cancers and healthy controls. Also, NK cell activity has shown to be decreased in cancer patients, as this may be indicator of susceptibility to disease.^[^ ^59^, ^60^, ^61^ ^]^ However, the patients we processed had undergone various cancer treatments, such as immunotherapy and anticancer treatment; results here might differ from previous studies mostly examining an initial state of cancer. The increase in concentration in NK‐derived EVs shown here may be linked to NK cell activation. Through various signaling pathways, such as dendritic cell activation, the presence of tumors in the body leads to an increase in NK cell activation.^[^ ^62^ ^]^ While some previous investigations have displayed no noticeable increase in NK cells^[^ ^59^, ^60^, ^61^ ^]^ or NK‐Exos^[^ ^57^, ^63^ ^]^ in cancer patients, these studies isolated bulk exosomes in samples, using less‐sensitive methods such as ultracentrifugation. In this study, we specifically isolate NK cells, and utilized secreted NK‐Exos for following studies. Thus, we believe that the exosomes recovered are NK cell‐derived and thus can more accurately define the specific concentration in patients compared to healthy donors. These results are supported by other lymphocytes, such as T cells, which demonstrate significant increases in exosome release once converted from resting to active states.^[^ ^64^ ^]^ While little investigation has been done into the cause of increased exosome release in activated NK cells or NK cells exposed to cancer, it is likely the correlation between cell activation and exosome release follows that of T cells. Thus, the higher incidence of NK cell activation in the NSCLC patients examined in this study likely leads to the increase in extracellular vesicle concentration in these patients compared to healthy donors, as seen in Figure 5d. The NK‐Exo secretion rate normalized by the number of NK cells captured on chip also confirmed that NK cells from lung cancer patients secrete more exosomes compared to that of healthy donors (Figure 5e). A size profiling of recovered NK‐Exos is described in Figure S7 (Supporting Information), implying healthy donors secrete more microvesicle‐like EVs, whereas cancer patients secrete smaller vesicles, although further studies are needed to verify this observation.

We also examined the cytotoxic potential of NK‐Exos from clinical samples on cancer cells. Same number of NK‐Exos from NSCLC cancer patients and healthy donors were applied to in‐house patient‐derived expanded CTC line and incubated for 72 h. Following incubation, the cells underwent live/dead fluorescent staining and microscopy scanning to identify live cells. As shown in Figure 5f, in‐house patient‐derived expanded CTC line were mostly killed by patient (*LC4*)‐derived NK‐Exos (center) compared to that without NK‐Exos (left) after 72 h. At the same time, the cancer cells were completely killed by NK‐Exos from *HD2* (right). Live cell number per well between control groups (without NK‐Exos) and NK‐Exo treated groups was significantly different (*p* < 0.0001).

We then compared NK‐Exo's cytotoxic capabilities between NSCLC patients and healthy donors (Figure 5g). At the same conditions, NK‐Exos from healthy donors show higher average specific lysis percentage (98.1%) than that of cancer patients (82.4%). This difference between two groups is significant (*p* = 0.0024). This implies that single NK exosomes from healthy donors have greater cytotoxic potentials; however, as previously noted, the NSCLC patients tested contained significantly more bloodborne NK exosomes than the healthy donors per mL of blood.

### 2.6 Correlation Study between NK Cell/Exosome and Circulating Tumor Cell Using Clinical Samples

**Figure 6:**
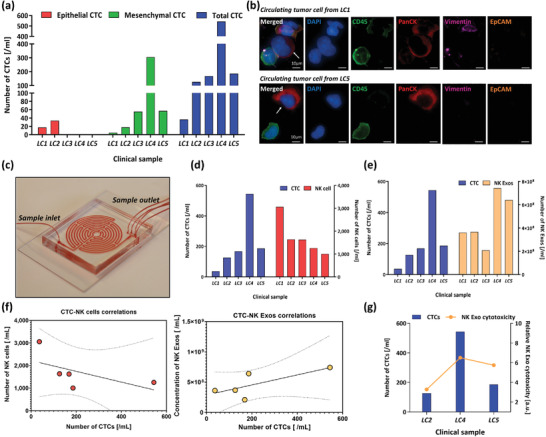
Profiling of circulating tumor cell (CTC) populations in non‐small cell lung cancer (NSCLC) patients and correlation with NK cells and NK cell‐derived exosomes: a) profiling in quantity of epithelial, mesenchymal, and total CTCs between NSCLC patients; b) representative images of CTCs recovered from the patients; c) image of label‐free CTC isolation platform; d) comparison between total CTC and NK cell number; e) comparison between total CTC and NK exosome concentration; f) correlation between CTC‐NK cell (left, *r* = −0.580, *P*‐value = 0.305) or CTC‐NK‐Exo (right, *r* = 0.732, *P*‐value = 0.159); g) correlation between relative total cytotoxicity of NK‐Exos and total CTC number in samples.

Five peripheral blood samples enrolled in clinical studies of NK cell/exosome were processed through our label‐free circulating tumor cell isolation platform to evaluate correlation between CTC numbers and NK cell/exosome concentrations (**Figure** **6**). These five patients were with metastatic, stage IV NSCLC patients. Among these five patients, there were patients with EGFR mutations (*n* = 3), ROS1 rearrangements (*n* = 1), and ALK fusion (*n* = 1). The patient information details are listed in Table S1 (Supporting Information). After CTC isolation, the pass‐through samples from outlet no. 2 were analyzed for CTCs as described previously.^[^ ^65^ ^]^ Overall CTC numbers for five patients are described in Figure 6a. CTCs were detected by IF staining. CTCs were defined and enumerated if they were PanCK+/CD45−/DAPI+. Furthermore, heterogeneous CTC populations, including CTCs expressing epithelial (EpCAM), mesenchymal (Vimentin), or both markers, were detected. Figure 6b illustrates IF staining of some isolated single/cluster CTCs stained positive for PanCK (red), EpCAM (orange), and Vimentin (Pink), and negative for CD45 (green) to distinguish CTCs from white blood cells (WBCs). We determined that all five patients had detectable CTCs with an average of 212 ± 194 total CTCs mL^−1^ (Figure 6a). Of the captured CTCs among all the patient samples (*n* = 5), 49 ± 54 were CTCs mL^−1^, 10 ± 15 were EpCAM+ CTCs, 88 ± 123 were Vimentin+ CTCs, and 65 ± 91 were double‐positive CTCs mL^−1^.

After this identification of CTCs, we compared CTC numbers to NK cells detected (Figure 6d) and NK‐Exo concentration (Figure 6e). From this correlation study, we discovered a negative correlation (*r* = −0.580, *P*‐value = 0.305) between NK cell number and CTC number (Figure 6f). This negative correlation between CTC number and NK number has previously been shown in a variety of cancers including NSCLC, breast, colorectal, and prostate cancer.^[^ ^66^, ^67^ ^]^ It is believed that a decrease in the presence of lymphocytes, including NK cells, provides limited immune response which enables tumor cell growth and allows for an increase in CTC concentration in blood. A relatively strong positive correlation (*r* = 0.732, *P*‐value = 0.159) was observed between NK‐Exo concentration and CTC number. To our knowledge, this finding has not been previously reported. This positive correlation could be a consequence of an increased presence of bloodborne CTCs leading to more circulating NK cells becoming stressed, which coupled with the proper environment, may induce the stressed NK cells to actively release more exosomes. This concept has been demonstrated with other immune cells, such as T cells, which release increased amounts of exosomes upon stress induced activation brought on by the presence of cancer.^[^ ^68^ ^]^ Larger investigations need to be done to have significant results. To evaluate individual samples’ NK‐Exo cytotoxicity with consideration of both NK‐Exo concentration and each NK‐Exo's cancer cell lysis performance, we prepared relative NK‐Exo cytotoxicity for three samples enrolled in the cytotoxicity study. This relative cytotoxicity value shows close correlation with CTC numbers in blood (Figure 6g). This result suggests that, while NK cell number in blood may not in itself affect the total cytotoxicity, the increase of NK‐Exos in bloodborne concentration brought about by NK activation with abundant CTCs greatly increases its antitumor capabilities.

## 3 Conclusion

As a proof of concept, here we have demonstrated the possibility of a streamlined microfluidic approach to on‐chip biogenesis and harvest of natural killer cell‐derived exosomes through comprehensive studies using NK cell lines and clinical samples from lung cancer patients. In the future, NK cell‐derived exosomes may find a complementary use as both diagnosis and therapeutic tools for patients with cancer. Given the burgeoning interest in this field, it is important to fill the technical gaps pertaining to exosome isolation, harvest, and expansion. We hereby present a highly sensitive method to isolate NK exosomes derived from viable NK cells using our NK‐GO chip. Using the NK‐GO chip, we showed that patients with non‐small cell lung cancer presented with high numbers of NK and NK cell‐derived exosomes compared with healthy donors. These concentrations were further correlated with numbers of bloodborne circulating tumor cell in each sample, and we found that a sample having a higher number of CTCs shows lower NK cells, but may lead to a greater secretion of NK cell‐derived exosomes. Furthermore, we were able to demonstrate functional relevance of NK cell‐derived exosomes as shown by their cytotoxic effects against in‐house patient‐derived expanded CTC line. The immediate future studies can focus on doing the similar analysis with large cohort of patient samples as well as healthy donors to make more robust comparisons. Future studies will be needed to further validate our initial observations and how best to use this to inform clinical needs. Given the critical diagnostic value of CTCs in cancer, immune‐phenotyping associated immune cells such as NKs may complement and enhance their predictive potential of patient prognosis. Moreover, such a precise isolation of cells may also help us investigate interactions between CTCs and immune cells in circulation, enabling the understanding of potential metastasis‐specific immune surveillance mechanisms. We expect that our versatile microfluidic platform can provide a foundation to be used for hitherto undiscovered roles of exosomes in cancer and other disease states.

## 4 Experimental Section

##### Cell Culture and Model Sample Preparation

NK‐92MI cells were cultured in minimum essential medium (MEM)‐alpha (Gibco, USA) containing 20% fetal bovine serum and 1% penicillin‐streptomycin solution. To prepare model samples for NK cells, NK‐92MI cells were prelabeled with a CellTracker dye with green (ThermoFisher, USA) and ≈10^3^–10^5^ NK cells were spiked into 1 mL of PBS buffer or whole blood. For NK exosome secretion experiments in well plates, 3 million NK cells were seeded into each 10 mm suspension cell culture dish (Corning, USA) with media containing exosome‐depleted serum (Gibco, USA). After 48 h of cell seeding, supernatant from 8 to 10 cell culture dishes was collected and centrifuged at 500 *g* for 5 min before the supernatant is again centrifuged at 12 000 *g* for 20 min. Model samples for NK cell‐derived exosomes were prepared by ultracentrifuging NK cell culture supernatant. After ultracentrifugation, the exosome concentration using NanoSight (Malvern, UK) was measured, and a known concentration of exosomes was spiked for model sample preparation.

##### Human Blood Sample Preparation

The blood sample collection and experiments were approved by Ethics committee (Institutional Review Board and Scientific Review Committee) of the University of Michigan. Informed consent was obtained from all participants of this clinical study, and the blood samples of cancer patients were obtained after approval of the institutional review board at the University of Michigan (HUM00119934). All experiments were performed in accordance with the approved guidelines and regulations by the ethics committee at the University of Michigan. The blood samples were used within 2 h of extraction, and each whole blood sample was directly processed by NK‐GO chip and Labyrinth for NK cell isolation and CTC isolation, respectively.

##### NK‐GO Chip Fabrication and Surface Modification

Graphene oxide microfluidic devices were used for the high‐purity isolation of rare cells.^[^ ^69^ ^]^ This device was improved and optimized for NK cell applications. Briefly, gold‐patterned silicon wafers were soaked in GO solution for 10 min, then bonded to a polydimethylsiloxane (PDMS) chamber using a corona discharge. NK‐GO chips were injected with N‐γ‐maleimidobutyryloxysuccinimide ester (GMBS) cross‐linker in ethanol and incubated for 30 min. The device was then washed with PBS and 500 µL of 10% NeutrAvidin in PBS was injected through the device. The prepared GO chips were conjugated with NK cell antibodies using avidin‐biotin affinity‐based immobilization. Biotinylated antibodies against CD56 were flowed through the device for NK cell capture.

##### NK Cell Isolation and On‐Chip NK Cell Exosome Harvesting

Before processing each sample containing NK cells, the device was blocked with a 3% BSA solution in PBS to minimize nonspecific binding. Cell capture was performed by flowing the sample containing NK cells through the devices using a syringe pump at a flow rate of 1 mL h^−1^. PBS buffer was then applied to wash away nonbonded cells. After cell isolation and washing, the cells were fixed with 4% PFA solution and permeabilized on the chip, followed by staining with 4′,6‐diamidino‐2‐phenylindole (DAPI) fluorescent dye for staining cell nuclei. The chips were then scanned under fluorescent microscope (Ti2, Nikon, Japan). After scanning, 300 µL of MEM‐alpha serum‐free media with 20% exosome‐depleted fetal bovine serum (FBS) (Gibco, USA) and 200U/ml recombinant human interleukin 2 (rhIL‐2) (PeproTech, USA) was then pumped into the device with low flow rate (1 mL h^−1^). Incubation for 12 h with the whole device was then performed at 37 °C, 5% CO_2_ incubator Galaxy 14S (Brunswick, USA).

##### On‐Chip NK‐92MI Cell Enumeration for Model Sample Experiments

The number of cells captured on the chip was obtained by counting CellTracker Dyed/DAPI+ cells on fluorescent images of the device, and the total cell number processed was obtained using different methods depending on background solutions. For the cells spiked in buffer, total cell number can be obtained by counting the cell number in the solution which flowed out of the chips. Under the principle of mass balance, total cell number is simply the sum of cells on chip and cells collected in the waste. When the cells were spiked in healthy blood, the total cell number was obtained by spiking same amount of cell solution into well plates to be counted.

##### Immunofluorescence Staining of Cells

To verify NK cells in clinical samples, the NK cell staining panel was optimized and set. For staining optimization, NK‐92MI and Jurkat cell slides were using a 10 min, 800 *g* spin cycle on a Cytospin 4 (ThermoFisher Scientific, USA), followed by a 10 min 4% PFA fixation. Cells were then permeabilized using 0.2% Triton X‐100 (Sigma‐Aldrich, USA) for 3 min, followed by three 5 min washes using phosphate buffer saline (Gibco, USA). The slides were then blocked with 10% goat serum (Invitrogen, USA) for 30 min. Following that, the primary antibodies were applied for 1 h at room temperature. After washing off the primary antibodies using another three 5 min PBS washes, secondary antibodies were applied and incubated for 30 min. Coverslips were then applied with ProLong Gold antifade reagent with DAPI (Molecular Probes, USA). Primary antibodies against CD56 (A7913, Abclonal, USA) and NCR1 (A14499, Abclonal, USA) and secondary antibody against rabbit IgG (A21245, ThermoFisher, USA) were included in the NK cell staining panel.

For staining on chip using clinical samples, 1 mL 4% PFA was flowed into the device after 12 h incubation and harvesting supernatant. After 40 min fixation, 1 mL PBS was used to wash off the PFA. The chips were permeabilized with 0.2% Triton for 30 min before blocking with a combination of 3% BSA (Gibco, USA) and 2% goat serum. Primary antibodies were then applied and incubated at 4 °C overnight. Secondary antibody was then flowed following a 2 mL PBS wash. The devices were then washed and applied with DAPI. The entire chip was then scanned under a multichannel fluorescent microscope (Nikon Ti2, Japan) for enumeration.

##### Preparation of ExoBeads for NK Exosome Capture and Release

For the isolation and release of the captured exosomes, desthiobiotinylated anti‐CD63 (Life Technologies Corporation, USA) was used. Binding chemistry of desthiobiotinylated (d‐biotin) anti‐CD63 to streptavidin is similar to biotinylated anti‐CD63, with the main difference involving binding affinities. The desthiobiotin‐streptavidin complex (*K*_d_ 10^−11^ m) can be broken and replaced by biotin‐streptavidin (*K*_d_ 10^−15^ m) interactions because of biotin's significantly higher binding affinity toward streptavidin.^[^ ^45^, ^46^ ^]^ Biotinylated anti‐CD63 was affixed to streptavidin‐conjugated magnetic beads, DynaBeads T1 (Invitrogen, Norway), as follows. First, 20–40 µL of 10 mg mL^−1^ streptavidin‐conjugated magnetic beads in solution were deposited into a small vial and washed with 0.2 µm filtered PBS multiple times. After that, three different dilution ratios of desthiobiotinylated anti‐CD63 (1:5, 1:10, and 1:20) solution in 1% BSA were applied to the magnetic beads. The beads with antibodies underwent a 1 h incubation period. After this incubation, the beads were washed three times with filtered PBS. In each case, a magnet was applied to the side of the vial to secure the beads while the solution was extracted. For the control beads, instead of antibody solution, only a 1% BSA solution without antibody reagent was used, and the remaining steps were followed the same.

##### NK Cell‐Derived Exosomes Isolation/Release Using Magnetic Beads

200 µL of supernatant from the device was directly applied to the prepared magnetic beads (ExoBead). This mixture was incubated on a rotator for an hour, and magnetic beads were separated using a magnet, followed by PBS washing three times. The beads right after this step underwent SEM or western blot analysis. For the release of the captured exosomes on the beads, 1 mL of 0.5 × 10^−3^ m biotin solutions was added to the separated beads, consisting of 1 mL of filtered water and 10 µL of biotin solution and incubated for 0.5–2 h. The biotin solution was placed in a new tube and analyzed by NTA analysis. In all cases, the effluents and resultants after bead removal underwent NTA analysis for verification.

##### Ultracentrifugation of NK Cell‐Derived Exosome

Ultracentrifugation was used for two reasons: 1) characterization of exosome secretion from cells in each well plate and 2) ExoBead device characterization. In both cases, a Sorvall ultracentrifuge (ThermoFisher, USA) was used. For comparison study, the same volume of initial plasma sample was used but diluted into 1 mL of PBS. Blood samples were first centrifuged at 2000 *g* for 15 min, and then 12 000 *g* for 20 min. After initial ultracentrifugation at 100 000 *g* for 90 min, the supernatant was aspirated and another 38 mL of PBS was injected for a second round of ultracentrifugation at the same conditions. The pellet after the second UC was gently spiked into 500 or 200 µL, for well‐plate samples and ExoBead characterization samples, respectively.

##### Field Emission Scanning Electron Microscopy (FESEM)

The capture and release of exosomes by ExoBeads was examined by FEI Nova 200 Nanolab Dualbeam FIB scanning electron microscope under beam energies (2.0–5.0 kV) at the Michigan Center for Materials Characterization at University of Michigan. Right after capture and release experiments, ExoBeads were immobilized on clean carbon tape and the specimen was naturally dehydrated. The dehydrated specimen was then mounted on an SEM stub and coated with gold by sputtering. The images from SEM were saved and processed using the desired SEM image analysis software.

##### Nanoparticle Tracking Analysis

Evaluation of exosome concentration and size distribution was analyzed by NTA using NanoSight NS300 (Marven Instruments, UK). 30 µL of the prepared solution was applied to the jig of the system, and laser module was mounted inside the main instrument housing. NTA visualizes the scattered lights from the particles of interest based on their Brownian motion. This movement was monitored through a video sequence for 20 s in triplicate. All data acquisition and processing were performed using NanoSight NS300 control software, and concentration of particles in exosome size range was used for calculating capture and release efficiencies of the present platform.

##### Protein Extraction and Western Blot Analysis

RIPA buffer with 1% protease inhibitor was prepared for lysis of captured exosomes. 30 µL of the prepared buffer solution was injected to postcapture ExoBeads and incubated for 20 min. After incubation, the protein lysate was aspirated and stored separately. Total amount of proteins in the lysate from the ExoBeads was measured by standard micro bicinchoninic acid (BCA) analysis according to the manufacturer's instructions. Western blot analysis was performed on a precast 4–20% SDS gel (BioRad, USA). The samples were prepared in 4× Laemelli buffer with 2‐mercaptoethanol and heated to 90 °C for 6 min before loading onto the gel. The gel was run at 120 V for 50 min before transferring at 120 V for 1 h on ice. Blocking was performed in 5% nonfat milk in tris‐buffered saline with 0.1% Tween® 20 detergent (TBST) for 90 min. Primary antibody incubated overnight on a rocker at 4 °C at a concentration of 1:1000 in 3% nonfat milk in TBST. Thorough rinsing was performed, and then secondary antibody was incubated for 90 min at room temperature at 1:1500 in 3% nonfat milk in TBST.

##### Isolation of Circulating Tumor Cells from NSCLC Patients Using Label‐Free Microfluidic Device

Briefly, 18 mL of blood was collected in ethylenediaminetetraacetic acid (EDTA) tubes and processed through the Labyrinth within 2 h of collection.^[^ ^65^ ^]^ Prior to processing in the Labyrinth, RBCs in the blood samples were removed using Ficoll‐Paque PLUS Media (GE Healthcare, USA) following the company's protocol. The supernatant (plasma and buffy coat layers), which included all whole blood components except RBCs, was carefully removed and diluted with PBS (1:5). The diluted samples were then processed through the Labyrinth at a flow rate of 2500 µL min^−1^. The pass through product from outlet was collected after stabilization.

##### NK Exosome Uptake and Cytotoxicity Experiment

NK‐92MI‐derived exosomes were prepared using UC of cell culture media. For uptake experiments, the exosomes were fluorescently labeled using PKH dye (Sigma‐Aldrich, USA) The exosomes were then added into 96‐well plates where patient‐derived expanded CTC line was seeded 24 h before. After 3 h of incubation, cancer cells were then fixed using 4% PFA for 10 min. Within the well plate, the cells were stained with Cellmask (ThermoFisher, USA) and DAPI for 10 min. The well was then imaged under a fluorescent microscope.

For evaluating potential cytotoxicity effects, NK cell‐derived exosomes were resuspended into serum‐free RPMI media after ultracentrifugation. Control wells were seeded in parallel with the same cell density (2000 cells per well). At day 0, 50 µL of serum‐free RPMI media and RPMI media containing exosomes were added into control wells and exosome wells. After 24, 48, and 72 h, a live/dead assay kit was performed under manufacturer's instruction (L3324, ThermoFisher, USA). The entire wells were scanned using a fluorescent microscope (Nikon, Ti2). The images were then counted using autorecognition of regions of interest (ROIs) in the Nikon software.

##### NK Exosome Cytotoxicity Experiment Using Clinical Sample‐Derived NK Exosomes

In‐house patient‐derived expanded CTC line was used for NK exosome cytotoxicity experiments. Briefly, CTCs from an EGFR mutant NSCLC (encoded R022‐V8) were isolated using Labyrinth, then they were expanded in vitro successfully.^[^ ^58^ ^]^ Given that in‐house patient‐derived expanded CTC line might resemble cancer cells in circulation than conventional cancer cell lines, this patient‐derived expanded CTC was used for evaluating cytotoxic potential of NK‐Exos. The cells were seeded into 384‐well plate at a density of 100 cells per well and incubated for 24 h before the event of NK‐Exo treatment. To prevent further contaminations from biotin and magnetic beads residues, recovered NK‐Exos from ExoBeads were ultracentrifuged and resuspended into serum‐free RPMI media. 1–2.3 × 10^7^ of NK‐Exos were seeded into the wells containing preseeded cancer cells. After 72 h of incubation, a live/dead kit‐based assay was performed under manufacturer's instruction (L3324, ThermoFisher, USA), followed by entire well scanning using a fluorescent microscope (Nikon, Ti2). The images were then counted using autorecognition of ROIs in the Nikon software.

##### Statistical Analysis

All results are presented as mean ± standard deviation. Statistical analysis was demonstrated using Graphpad Prism 9. Unpaired *t*‐tests (two‐tailed) were used to compare the differences between live cell count between NK exosome treated (*n* = 4) versus control (*n* = 4). Statistical significance was defined as *p* < 0.05.

## Conflict of Interest

S.N. is one of the named inventors on a patent for Microfluidic Labyrinth Technology granted to the University of Michigan. S.N. is also the co‐founder of Labyrinth Biotech Inc. The funders and the company had no role in the design of the study; in the collection, analyses, or interpretation of data; in the writing of the manuscript; or in the decision to publish the results.

## Supporting information

Supporting Information
