# Supplementary material for: On‐Chip Biogenesis of Circulating NK Cell‐Derived Exosomes in Non‐Small Cell Lung Cancer Exhibits Antitumoral Activity
Source: Adv Sci (Weinh). 2021 Jan 28;8(6):2003747. doi: 10.1002/advs.202003747 (PMC7967048; doi:10.1002/advs.202003747)
Supplement: Supplementary file 1 — Supporting Information [file ADVS-8-2003747-s001.pdf]

© 2020 Wiley-VCH GmbH

ADVANCED SCIENCE
Open Access

Supporting Information

for *Adv. Sci.,* DOI: 10.1002/advs.202003747

On-Chip Biogenesis of Circulating NK Cell-Derived Exosomes in Non-Small Cell Lung Cancer Exhibit Anti-Tumoral Activity

Yoon-Tae Kang,, Zeqi Niu, Thomas Hadlock, Emma Purcell, Ting-Wen Lo, Mina Zeinali[1], Sarah Owen, Venkateshwar G. Keshamouni, Rishindra Reddy, Nithya Ramnath, and Sunitha Nagrath**

WILEY-VCH

DOI: 10.1002/ ((please add manuscript number))

Supplementary Information

On-Chip Biogenesis of Circulating NK Cell-Derived Exosomes in Non-Small Cell Lung Cancer Exhibit Anti-Tumoral Activity

Yoon-Tae Kang[1, ¶], *Zeqi Niu*[1, ¶], *Thomas Hadlock*[1], *Emma Purcell*[1], *Ting-Wen Lo*[1], *Mina Zeinali*[1], *Sarah Owen*[1], *Venkateshwar G. Keshamouni*[3], *Rishindra Reddy*[4], *Nithya Ramnath*[2, *] *and Sunitha Nagrath*[1, *]

[1]Department of Chemical Engineering, University of Michigan, Ann Arbor, MI 48109, USA

Department of Chemical Engineering, Biointerface Institute, and Rogel Cancer Center, University of Michigan, 2800 Plymouth Road, NCRC B10-A184, Ann Arbor, MI 48109, USA
E-mail: snagrath@umich.edu

[2]Department of Internal Medicine, University of Michigan, Ann Arbor, MI 48109, USA
E-mail: nithyar@med.umich.edu

[3]Michigan Medicine, Pulmonary & Critical Care Division, University of Michigan, Ann Arbor, MI, 48109, USA

[4]Michigan Medicine Thoracic Surgery Clinic, Taubman Center, 1500E Medical Center Dr. SPC 5344, Ann Arbor, MI, 48109, USA

[¶] These authors contributed equally to this work
[*] Co-corresponding authors

Contents

S1. Evaluation criteria for the present devices and definitions 3

S2. NK cell derived exosome harvesting off and on NK-GO chip 6

S3. Immunofluorescence staining of NK cells and Jurkat cells using NK staining panel 7

S4. Cellular uptake of extracellular vesicles from Natural Killer cells 8

S5. Evaluation of cytotoxicity of NK-92®MI derived exosomes 9

S6. Size profiling of NK-Exos from clinical samples 11

S7. Patients information 12

S8. Comparison between conventional EV isolation and current platform 13

Figures & Table

Fig. S1. Comparison of exosome secretion and purity of secreted vesicles from off-chip and on-chip culture for 12 hours 6

Fig. S2. Immunofluorescence staining of NK-92®MI cells 7

Fig. S3. Immunofluorescence staining of Jurkat cell line 7

Fig. S4. Cellular uptake of NK Exosomes 8

Fig. S5. Cytotoxicity evaluation of residual biotin solution 9

Fig. S6. Optimal NK exosome concentration for cytotoxicity experiments 10

Fig. S7. Size profiling of recovered extracellular vesicles from clinical samples 11

Table S1. Clinical information of the patient samples 12

S1. Evaluation Criteria for the Present Devices and Definitions

Capture efficiency is the fraction of the cells or exosomes isolated by NK-GO chip or ExoBeads, compared to the initial amount of spiked cells or exosomes, respectively. NK cell capture efficiency was calculated as follows.

$$\boldsymbol{Capture\ efficiency}\ (\%) = \frac{cell\ number\ captured\ on\ chip}{total\ cell\ number\ processed} \times 100\%$$

For exosomes, the amount captured was evaluated by subtracting the effluent concentration from the initial concentration. For each sample, we only evaluated the amount of exosome like vesicles ranging 30-150nm, and it is calculated as follow.

$$\boldsymbol{Capture\ efficiency}\ (\%) = \left[1 - \frac{(concentration\ of\ exosome - sized\ vesicles\ in\ capture\ effluent)}{(concentration\ of\ exosome - sized\ vesicles\ in\ initial\ sample)}\right] * 100\%$$

Release efficiency is the fraction of the exosomes released from ExoBeads compared to the total amount of isolated exosomes. To evaluate this, we additionally measured the concentration of released sample after biotin incubation, and compared this concentration to the amount of captured exosomes, which has been calculated as previously described. Thus, release efficiency is calculated as follow.

$$\textbf{Release Efficiency}\ (\%) = \left[\frac{(concentration\ of\ exosomes\ in\ release\ resultant)}{(con.of\ exosomes\ in\ initial - con.of\ exosomes\ in\ capture\ effluent)}\right] * 100\%$$

In order to evaluate whether the present device captures purified exosomes from the heterogeneous samples, we calculated the purity of the sample, the fraction of the concentration in exosome sized vesicles compared to whole concentration. Whole concentration values came directly from NTA result, and the concentrations of exosome sized vesicles were re-calculated from the NTA raw data.

$$\textbf{Purity}\ (\%) = \left[\frac{(concentration\ of\ exosome - sized\ vesicles\ in\ sample)}{(concentration\ of\ whole\ vesicles\ in\ sample)}\right] * 100\%$$

The recovery rate is the fraction of release resultant to the sum of the capture effluent and release resultant. If we do not know the initial concentration of sample but want to know the isolation tendency of the present device, a simpler version of capture/release efficiency is 'recovery rate' derived only from capture effluent and release resultant concentrations. It is calculated as follow.

$$\textbf{Recovery Rate}\ (\%) = \left[\frac{(concentration\ of\ exosome - sized\ vesicles\ in\ release\ resultant)}{(concentration\ of\ exosome - sized\ vesicles\ in\ capture\ effluent + release\ resultant)}\right] * 100\%$$

Particle-size distribution (PSD) analysis was used to profile our NK-Exo from clinical samples. The PSD is a list of values of a mathematical function that defines the relative number of particles present according to size. This includes the particle size span width, *D10*, *D50* and *D90*, as known

as three point specification or *D-value*. More specifically, those three *D-values* indicate the diameter of the particle at 10%, 50% and 90% of the cumulative distribution. For example, if *D50* is 150nm, it means that 50% of the particles in the sample from NTA are bigger than 150 nm and another 50% are smaller than 150nm.

S2. NK cell derived exosome harvesting off and on NK-GO chip

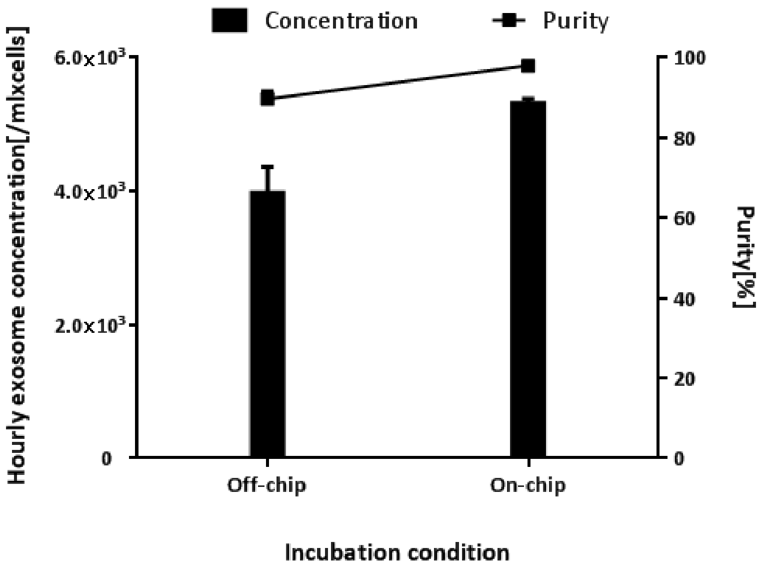

Figure S1. Comparison of exosome secretion and purity of secreted vesicles from off-chip and on-chip culture for 12 hours.

S3. Immunofluorescence staining of NK cells and Jurkat cells using NK cell staining panel

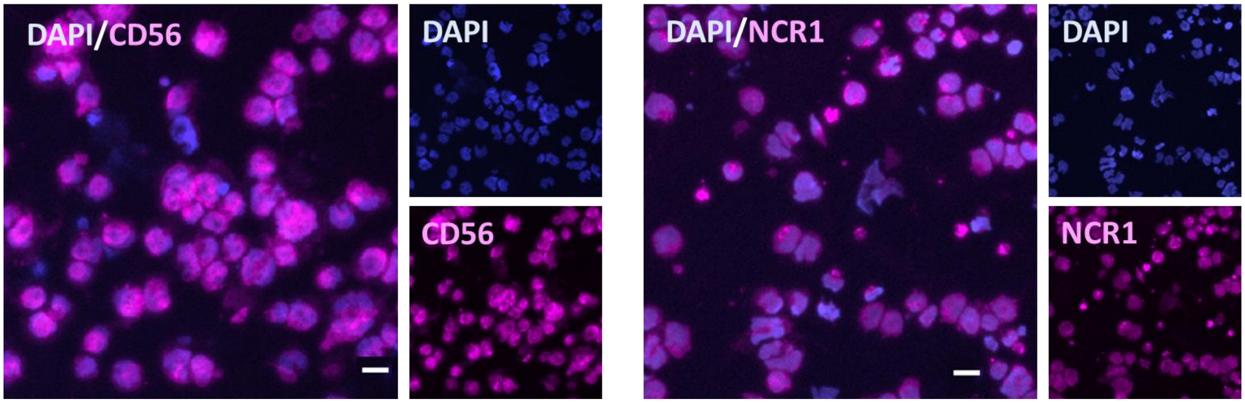

Figure S2. Immunofluorescence staining of NK92mi cells (Scale bar= 20μm)

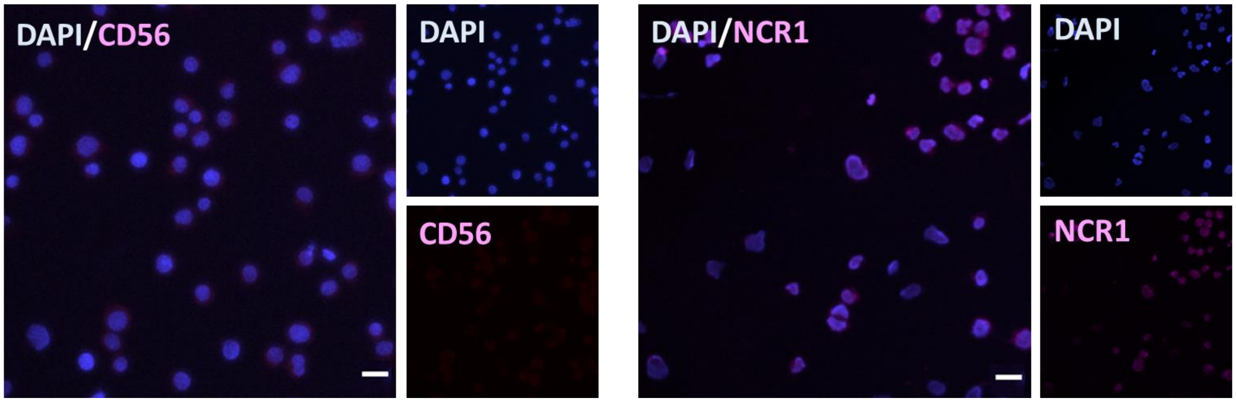

Figure S3. Immunofluorescence staining of Jurkat cell line (Scale bar= 20μm)

S4. Cellular uptake of extracellular vesicles from Natural Killer cells

NK92mi derived exosomes were harvested 24hrs after switching into serum free media using ultracentrifuge. In house CTC-pt2 line was plated onto a cytospin (ThermoScientific, USA) coverslip and kept in RPMI media 24hr before the day of imaging. Image was taken using Nanolive (Nanolive, Switzerland) 3D cell explorer.

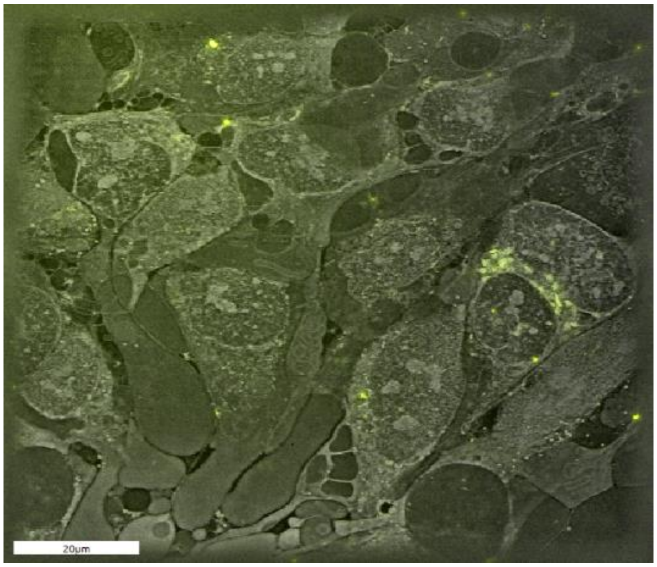

Figure S4. Cellular uptake of NK-Exosomes

S5. Evaluation of the parameters in NK-92®MI derived exosomes cytotoxicity experiments

S5.1. Effect of residual biotin

Initial cytotoxicity evaluation of NK92mi cell line derived EVs and biotin solution was done using CellTiter-Glo reagent (Promega®, USA). NK92mi derived EVs were incubated with serum-free MEM-alpha media for 24 hrs and then harvested using ultracentrifuge. In house CTC line was plated onto 96 well plate 24 hrs before adding treatments with a density of 1000 cells/well. NK92mi derived EVs are diluted into 3 different concentrations, 3.11×10^9 exo/mL, 3.11×10^8 exo/mL, 3.11×10^7 exo/mL with serum free RPMI media. In order to test the confounding effect biotin solution has on cancer cells, 4 different concentrations of biotin solution were also tested, and triton X-100 (Triton) was used as a negative control. Due to time limitations, this experiment was not repeated. We evaluated the effect of possible residual biotin from the releasing process after streamlined NK cell isolation and exosome secretion. As shown, the luminescence intensity correlates with cell viability, after 6 hrs, the presence of biotin does not decrease target cells' viability.

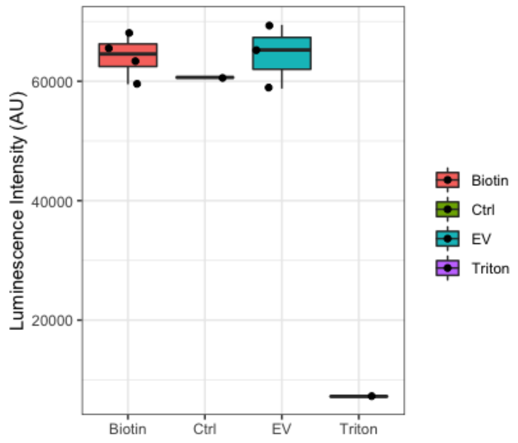

Figure S5. Cytotoxicity evaluation of residual biotin solution

S5.2. Optimal NK exosome concentration

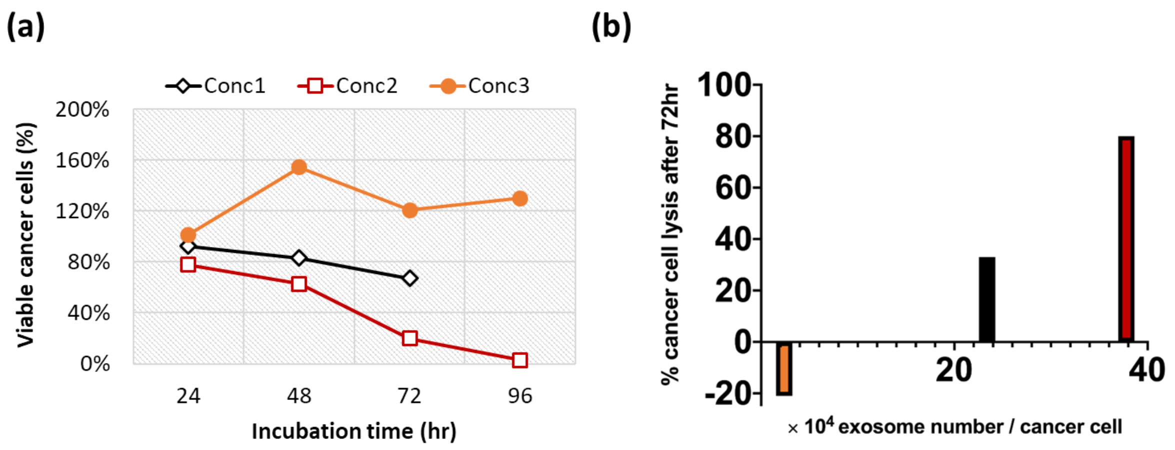

Figure S6. Optimal NK exosome concentration for cytotoxicity experiments

Three different conditions were applied when testing out NK exosomes' cytotoxicity effect (Figure S6). In experiment 1, in house derived CTC-pt1 line was seeded in 96 well plate with a concentration 2000 cells/well. 24 hrs after seeding, NK92mi derived exosomes were then applied with a concentration 2.34×10^5 exosomes per cell. In experiment 2, in house derived CTC-pt1 line was seeded in 384 well plate with a concentration 100 cells/well. 24 hrs after seeding, NK92mi derived exosomes were then applied with a concentration 3.78×10^5 exosomes per cell. 2000 cells/well were seeded in experiment 3. 24 hrs after seeding, NK92mi derived exosomes were then applied with a concentration 2.40×10^4 exosomes per cell. From the comparison of the two conditions, lower concentration (example 2.40×10^4 exosomes per cell) will facilitate the proliferation of cancer cell, while high concentration (example higher than 2.34×10^5 exosomes per cell) has cytotoxic effects.

S6. Size profiling of NK-Exos from clinical samples

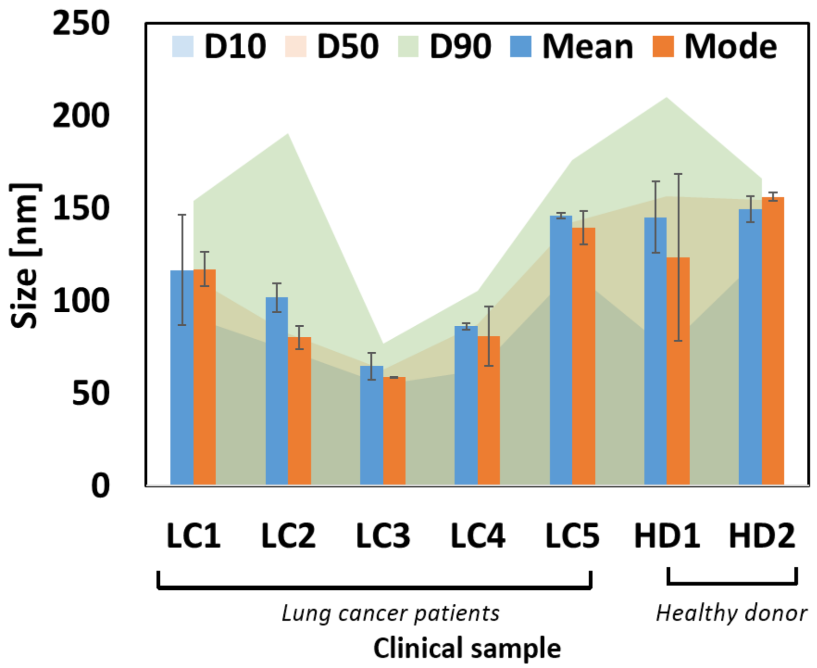

Figure S7. Size profiling of recovered extracellular vesicles from clinical samples in terms of mean, mode, and *D*-values (*D10*, *D50*, and *D90*).

S7. Patient information

Table S1. Clinical information of the patient samples

Cancer Type	Sample ID	Sample description						
		Sex	Age	Stage	Adenocarcinoma subtype	Mutation	Treatment	Days between blood draw and treatment start
Lung cancer patient	***LP1***	Female	45	IV	EGFR	EGFR, Exon 19 deletion, PDL1 10%	Osimertinib	399
	LP2	Female	55	IV	ROS1	ROS1 Mutation	Crizotinib	1022
	LP3	Male	66	IV	EGFR	EGFR Exon 19 deletion	Osimertinib	0
	LP4	Male	70	IV	EGFR	EGFR Exon 19 deletion	Osimertinib	175
	LP5	Male	60	IV	ALK	ALK translocation	Alectinib	1610
Healthy donor	***HC1***	Female	25	NA	NA	NA	NA	NA
	HC2	Male	32	NA	NA	NA	NA	NA

S8. Comparison between conventional exosome isolation and current platform [S1, S2]

	Ultracentrifugation	PEG-based kit	Exosome microfluidics		NK-GO chip+ExoBeads
**Purpose**	Exosome isolation	Exosome isolation	Exosome isolation		Specific on-chip exosome biogenesis/purification
**Principle**	Density, size, shape	Solubility/dispersibility of exosomes in water-excluding polymers	Immunoaffinity (protein expression)	Physical principle (Size/electrophoresis)	Immunoaffinity
**Specific Exo-subset recovery**	N/A	N/A	Partially available (biochemical principle)	Difficult (physical principle)	Available
**Working volume**	~30 ml	250 serum/plasma (ExoQuick ultra)	>300µl serum/media		200µl of NK-GO chip supernatant
**Processing time**	5-10 hours	0.5-12 hours	2-6 hours		3 hours
**Exosome yield**	Low	Low	High		High
**Exosomal Purity [S2]**	89.6 ± 6.5	72.9 ± 3.2	47.3 ± 14.0		82.1 ± 6.1
**Application**	Downstream analysis (protein, nucleic acids), NTA, exosome uptake experiments, etc.	Downstream analysis (protein, nucleic acids)	Downstream analysis (protein, nucleic acids), NTA, exosome uptake experiments, etc.		Downstream analysis (protein, nucleic acids), NTA, exosome uptake experiments, etc.

[S1] Zhang, P., Samuel, G., Crow, J., Godwin, A.K., Zeng, Y., Molecular assessment of circulating exosomes toward liquid biopsy diagnosis of Ewing sarcoma family of tumors. Transl. Res. 201, 136-153 (2018)

[S2] Kang, Y.T., Purcell, E., Palacios-Rolston, C., Lo, T.W., Ramnath, N., Jolly, S., Nagrath, S., Isolation and profiling of circulating tumor-associated exosomes using extracellular vesicular lipid-protein binding affinity based microfluidic device. Small 15(47), e1903600 (2019)
